# Assessing Engagement With Patient-Generated Health Data Recording and Its Impact on Health Behavior Changes in Multicomponent Interventions: Supplementary Analysis

**DOI:** 10.2196/35471

**Published:** 2022-05-03

**Authors:** Kaori Kinouchi, Kazutomo Ohashi

**Affiliations:** 1 Department of Children and Women's Health Area of integrated Health and Nursing Science Osaka University Graduate School of Medicine Suita, Osaka Japan; 2 Faculty of Global Nursing Otemae University Osaka Japan

**Keywords:** patient-generated health data, engagement, health behavior change, postpartum women, health data, health informatics, pelvic health

## Abstract

**Background:**

The use and sharing of patient-generated health data (PGHD) by clinicians or researchers is expected to enhance the remote monitoring of specific behaviors that affect patient health. In addition, PGHD use could support patients’ decision-making on preventive care management, resulting in reduced medical expenses. However, sufficient evidence on the use and sharing of PGHD is lacking, and the impact of PGHD recording on patients’ health behavior changes remains unclear.

**Objective:**

This study aimed to assess patients’ engagement with PGHD recording and to examine the impact of PGHD recording on their health behavior changes.

**Methods:**

This supplementary analysis used the data of 47 postpartum women who had been assigned to the intervention group of our previous study for managing urinary incontinence. To assess the patients’ engagement with PGHD recording during the intervention period (8 weeks), the fluctuation in the number of patients who record their PGHD (ie, PGHD recorders) was evaluated by an approximate curve. In addition, to assess adherence to the pelvic floor muscle training (PFMT), the weekly mean number of pelvic floor muscle contractions performed per day among 17 PGHD recorders was examined by latent class growth modeling (LCGM).

**Results:**

The fluctuation in the number of PGHD recorders was evaluated using the sigmoid curve formula (*R*^2^=0.91). During the first week of the intervention, the percentage of PGHD recorders was around 64% (30/47) and then decreased rapidly from the second to the third week. After the fourth week, the percentage of PGHD recorders was 36% (17/47), which remained constant until the end of the intervention. When analyzing the data of these 17 PGHD recorders, PFMT adherence was categorized into 3 classes by LCGM: high (7/17, 41%), moderate (3/17, 18%), and low (7/17, 41%).

**Conclusions:**

The number of PGHD recorders declined over time in a sigmoid curve. A small number of users recorded PGHD continuously; therefore, patients’ engagement with PGHD recording was low. In addition, more than half of the PGHD recorders (moderate- and low-level classes combined: 10/17, 59%) had poor PFMT adherence. These results suggest that PGHD recording does not always promote health behavior changes.

## Introduction

### Background

According to the Office of the National Coordinator for Health Information Technology, patient-generated health data (PGHD) is defined as health-related data created, recorded, or gathered by or from patients (or family members or other caregivers) to help address a health concern [1,2]. In 2018, the term PGHD was introduced into the Medical Subject Headings thesaurus [3]—a controlled and hierarchically organized vocabulary produced by the National Library of Medicine for indexing, cataloging, and searching of biomedical and health-related information—and the term became widely known. PGHD in paper form have been used in the past, but the development of digital health innovations has enabled the collection of a large amount of electronic PGHD easily through mobile phones, wearable devices, and several types of sensors. The use and sharing of PGHD by clinicians or researchers is expected to not only enhance the remote monitoring of specific behaviors that affect patient health, but also support patients’ decision-making on preventive care management, resulting in reduced medical expenses [4]. However, sufficient evidence on the use and sharing of PGHD in clinical settings is lacking [5], and the impact of PGHD recording on health behavior changes remains unclear [6-8]. Previous studies have incorporated PGHD techniques into multicomponent interventions. A scoping review [9] reported that multicomponent interventions used the following techniques to motivate users for PGHD recording: the provision of rewards and incentives, goal setting, reminders, feedback, social support, and entertainment elements such as gamification. Given the complexity and diversity of multicomponent interventions, it is difficult to evaluate the effect of PGHD recording, and there is no conclusive evidence that it improves health behavior [9,10].

### Our Previous Study

We conducted multicomponent interventions with reminder emails for pelvic floor muscle training (PFMT) to manage urinary incontinence (UI), in which users manually recorded the number of pelvic floor muscle contractions (PFMCs) performed as PGHD [11]. Daily reminder emails for PFMT were sent to the postpartum women’s smartphones for 8 weeks, and the number of PFMCs performed was recorded on a website via smartphone. Our results showed that this multicomponent intervention improved PFMT adherence (implementation rate and the number of times implemented per day and per week) and reduced the incidence of UI [11]. PFMT is a simple exercise to strengthen the muscles of the pelvic floor—participants contract and relax the pelvic floor muscle repeatedly—and it effectively treats and prevents UI [12,13]. However, it is difficult to maintain PFMT adherence in patients with only verbal instructions and leaflets [14,15]. Although our multicomponent intervention was expected to improve PFMT adherence, problems arose because the participants were not recording the number of PFMCs performed as PGHD every day as instructed by the researchers.

### Additional Rationale for This Study

Many users of digital behavior change interventions (DBCIs) that use technologies such as the internet, telephones, mobile phones, and environmental sensors [16] do not use these technologies as intended by researchers, and the number of users declines over time [17,18]. The attrition of DBCI users may impact the effectiveness of the interventions. Therefore, “effective engagement” that is sufficient to achieve the intended outcomes should be established [16,19]. Engagement with DBCIs is conceptualized as two synthetic constructs, “engagement as behavior” (eg, the extent of use of DBCIs or their components) and “engagement as subjective experience” (eg, intrinsic interest and enjoyment), and it is a dynamic process that is expected to vary both within and across individuals over time [20-22]. A scale for assessing engagement with DBCI has recently been developed [20,21]. It has been suggested that, by assessing engagement, researchers can determine when and how to tailor interventions to the individual, supplement with human support when needed, and identify the components required for intervention design [19,23].

### Goal of This Study

We conducted a supplementary analysis of the PFMC data stored on the server of our previous study [11]. This study aimed to assess patients’ engagement with PGHD recording (ie, “engagement as behavior”) and participants’ usability of PGHD recording (ie, “engagement as subjective experience”). Furthermore, we aimed to examine the impact of PGHD recording on health behavior changes in PFMT adherence among PGHD users who recorded their PFMCs consistently using latent class growth modeling (LCGM).

## Methods

### Recruitment and Sample

The participants were postpartum women who had delivered from January to August 2014 at an obstetric clinic in Osaka Prefecture, Japan, which performs approximately 600 deliveries per year, and had been assigned to the intervention group of our previous study [11]. The inclusion criterion of our previous study [11] was postpartum women with a smartphone and the exclusion criteria were participants with a history of pelvic surgery and cerebral infarction, as well as those with current hypertension, diabetes, hemorrhage, cystitis, neurological disease of the urinary system, chronic cough, and diuretic use. For postpartum care, a midwife provided the participants with verbal instructions on how to perform PFMT as detailed in a leaflet. The PFMT regimen included 3 sets of 6 PFMCs every day (ie, a total of 18 PFMCs per day), and the training duration was at least 8 weeks.

### Data Inclusion and Exclusion

This study used the data of 47 postpartum women who had been assigned to the intervention group of our previous study [11] for managing UI. There were no exclusion criteria, and no data were excluded from analyses.

### Study Design

This research is a supplementary analysis of our previous study, which improved PFMT adherence and reduced the number of postpartum women with UI. A detailed description of the study has been published in full [11]. In our previous study, participants received PFMT reminder emails via smartphone every day for 8 weeks, which contained a URL link to a website for manually recording the number of PFMCs performed.

### Ethics Approval

This study uses data collected during our previous study [11], which was approved by Osaka University Medical Science Department of Health Ethics Committee (approval number 268).

### Evaluation Outcomes

Data collected by the above procedure were classified into the following four categories: (1) participants’ demographic characteristics, including age, BMI before pregnancy, weight gain during pregnancy, and their child’s birth weight; (2) the number of participants who recorded their PGHD (ie, PGHD recorders); (3) each participants’ status of PGHD recording; and (4) weekly mean number of PFMCs performed per day among those who recorded it continuously. The participants’ usability of PGHD recording was evaluated after the 8-week intervention period with the question “Was it difficult to record the PFMCs every day?” The participants responded on a 5-point Likert scale (“Strongly agree,” “Agree a little,” “Neither agree nor disagree,” “Disagree a little,” or “Strongly disagree”). Furthermore, comments on the participants’ usability of PGHD recording were collected with the prompt: “Please comment on your experience of recording PFMCs on our system in the free-text field.” Participants voluntarily answered these two questions about usability. These data were encrypted using Secure Sockets Layer to prevent leakage of personal information during transmission and were stored on the server through the website. All data were downloaded in .csv format.

### Statistical Analysis

The participants’ demographic characteristics were described as continuous variables (reported as median values with IQRs) and categorical variables (reported as number of cases with percentages). To assess engagement with PGHD recording during the intervention period, a graph was plotted with the number of PGHD recorders on the y-axis and the number of days on the x-axis, and the approximate equation of the curve was calculated. To visualize each participant’s status of PGHD recording during the intervention period, a figure was created with gray-shaded cells indicating the days that a given participant recorded PGHD and the numbers within cells denoting how many PFMCs were performed that day. On the y-axis, participants are arranged based on the total number of times that PFMCs were recorded and the total number of PFMCs performed during the intervention period. Based on the lower asymptote that was obtained from the approximate curve, 17 participants (IDs 1-17) were classified as the high-engagement group and the remaining participants (IDs 18-47) were classified as the low-engagement group. Fisher exact test and Mann-Whitney *U* test were used to examine the differences in each group. To evaluate PFMT adherence among the 17 participants in the high-engagement group, the weekly mean number of PFMCs performed per day was calculated. To determine the model fit, we employed entropy and the Bayesian Information Criterion, and to determine the model number of the weekly mean number of PFMCs performed per day, we employed the Lo-Mendell-Rubin likelihood ratio test and the bootstrap likelihood ratio test [24,25]. LCGM is a statistical method that uses specific combinations of observed variables and can be used to identify groups of people with similar characteristics. Additionally, LCGM can determine individual phenotypes by identifying subgroups that follow similar trajectories over time [26]. LCGM in eHealth research is commonly used to determine potential trajectories and groups of engagement with DBCIs [27-33]. In this study, we used LCGM to determine PFMT adherence. The usability of PGHD recording was determined by organizing the participants’ comment data into qualitatively and inductively meaningful groups and calculating the number of cases and percentages for each group. A *P* value of <.05 was considered statistically significant for all analyses. LCGM analyses were performed using Mplus (version 8.6; Muthen & Muthen). Other analyses were conducted using JMP PRO software (version 15.1.0; SAS Institute Inc).

## Results

### Participants’ Demographic Characteristics

The participants’ demographic characteristics are shown in Table 1. The percentage of participants with UI at the baseline was 6% (3/47). The median age of the participants was 34 (IQR 31-36) years and 70% (33/47) were multiparous women. For BMI before pregnancy and weight gain during pregnancy, 72% (34/47) and 57% (27/47) of the participants were in the normal range, respectively. For child’s birth weight, 98% (46/47) of the participants reported birth weight of less than 4000 g.

**Table 1:** Comparison of demographic characteristics of participants having high and low engagement with patient-generated health data recording.

Characteristic			Total participants (N=47)		Engagement with PGHD^a^				*P* value
					High (n=17)		Low (n=30)		
UI^b^ at baseline, n (%)			3 (6)		1 (2)		2 (4)	.99^c^	
Age (years), median (IQR)			34 (31-36)		34 (32-37)		33 (30-36)	.21^d^	
Multipara, n (%)			33 (70)		14 (30)		19 (40)	.20^c^	
BMI before pregnancy (kg/m^2^), median (IQR)			20 (19-21)		20 (18-21)		20 (19-21)	.96^d^	
Weight gain during pregnancy (kg), median (IQR)			10 (8-12)		10 (9-12)		11 (89-12)	.89^d^	
Child’s birth weight (g), median (IQR)			3104 (2760-3384)		2885 (2736-3196)		3160 (2945-3480)	.05^d^	

^a^PGHD: patient-generated health data.

^b^UI: urinary incontinence.

^c^Fisher exact test.

^d^Mann-Whitney *U* test.

### Engagement With PGHD Recording

**Figure 1:**
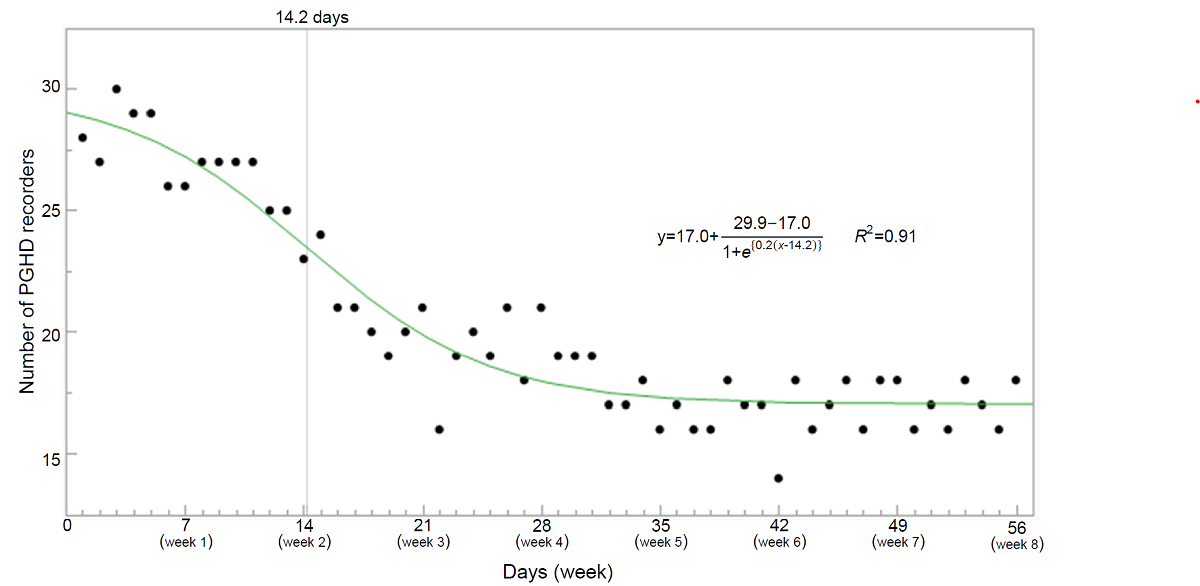
Engagement with patient-generated health data recording.

Engagement with PGHD recording is shown in Figure 1. The number of PGHD recorders was the highest at 3 days after the start of the intervention (31/47, 66%) and the lowest at 42 days (14/47, 30%). The approximate curve of the number of PGHD recorders (*y*) and the days (*x*) during the intervention period was calculated by the following sigmoid curve formula:




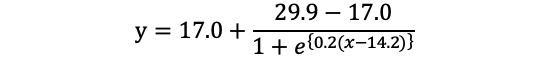




In the approximate curve, there was an inflection point at 14.2 days (95% CI 11.1-17.3; *P*<.001), with the upper asymptote at 29.9 participants (95% CI 27.2-32.6; *P*<.001) and the lower asymptote at 17.0 participants (95% CI 16.4-17.6; *P*<.001), and an *R*^2^ value of 0.91. The percentage of PGHD recorders during week 1 of the intervention was constant at 64% (30/47) and then decreased rapidly from week 2 to week 3. After week 4, 36% (17/47) of the participants continued to record the number of PFMCs performed until the end of the intervention.

**Figure 2:**
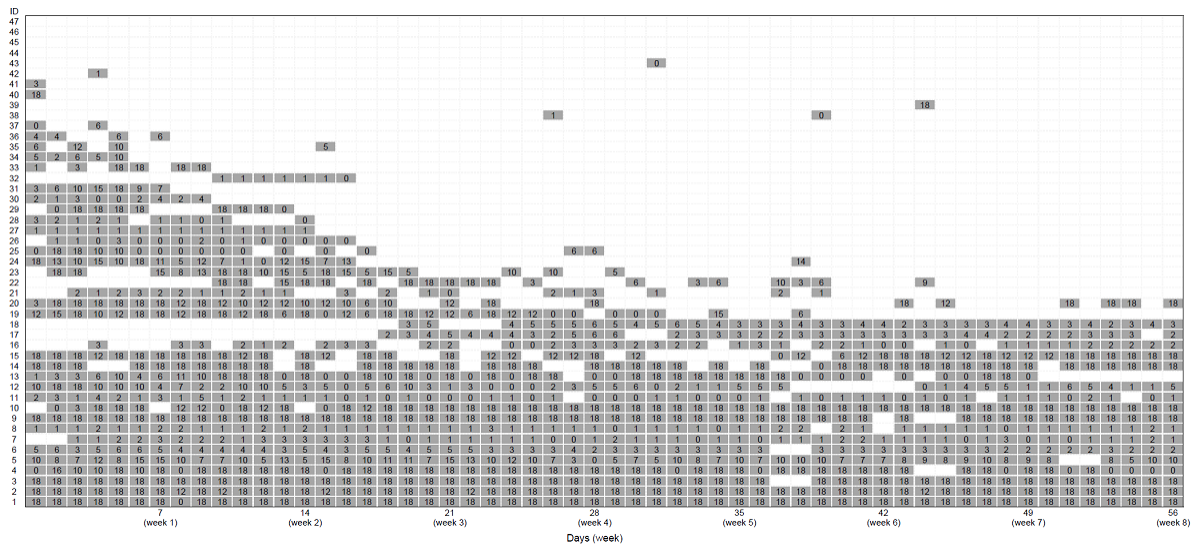
Status of patient-generated health data recording.

Figure 2 shows each participant’s status of PGHD recording. IDs 1 and 2 (2/47, 4%) completed PFMC recording every day. Conversely, IDs 44-47 (4/47, 9%) never recorded any data. High engagement with PGHD recording was observed for IDs 1-17 (17/47, 36%) and low engagement was observed for IDs 18-47 (30/47, 64%). A low number of participants recorded their PFMCs consistently. No significant difference was observed in baseline UI, age, birth history, BMI before pregnancy, weight gain during pregnancy, and birth weight between the two groups (Table 1).

### The Impact on Health Behavior Changes

Table 2 shows the model information obtained through LCGM for the PFMT adherence of the high-engagement group. The value of entropy of the 2-class model was 0.911 and that of the 3-class model was 1.000, indicating that the high-engagement group could be subdivided into 2 or more groups. No significant difference was found in the Lo-Mendell-Rubin likelihood ratio test and the bootstrap likelihood ratio test; however, the Bayesian Information Criterion was small, indicating a better fit in the 3-class model than the 2-class model. Finally, the 3-class model was selected. A 4-class model could not be produced.

**Figure 3:**
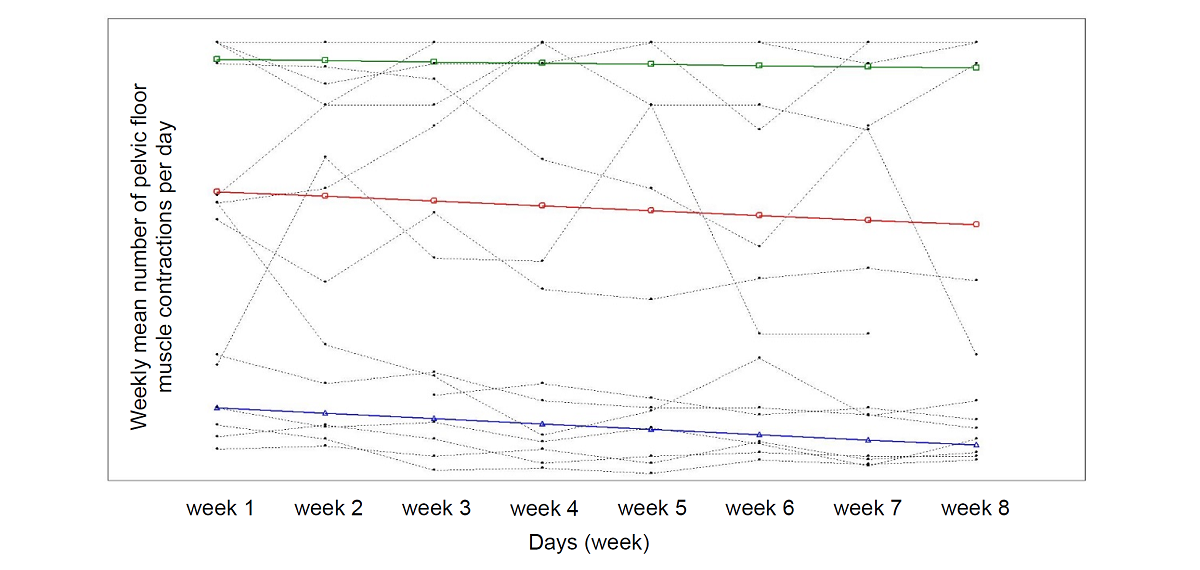
Pelvic floor muscle training adherence levels among patient-generated health data recorders. Green line: high; red line: moderate; blue line: low.

The 3-class model produced latent trajectories that corresponded to the weekly mean number of PFMCs performed per day. The following groups were defined: “high” for PGHD recorders who started with high PFMT adherence levels (7/17, 41%; Figure 3); “moderate” for PGHD recorders who started with moderate PFMT adherence levels (3/17, 17.6%; Figure 3); and “low” for PGHD recorders who started with low PFMT adherence levels (7/17, 41%). Of the 17 participants who continued to record data until the end of the intervention, 10 participants were in the moderate- and low-adherence groups, indicating that overall PFMT adherence was poor. Table 3 shows the characteristics of the 3 groups by PFMT adherence level.

**Table 2:** Model information by number of classes obtained through latent class growth modeling.

Test		Number of classes	
		2	3
Percent per class		41/59	41/18/41
Entropy		0.991	1.000
Bayesian Information Criterion		592.6	590.0
**Lo-Mendell-Rubin likelihood ratio test**		–285.6	–270.8
	*P* value	.43	.18
**bootstrap likelihood ratio test**		–288.6	–270.8
	*P* value	.29	.17

**Table 3:** Characteristics of the 3 groups by PFMT^a^ adherence level.

Characteristic		PFMT adherence level				*P* value
		High (n=7)	Moderate (n=3)	Low (n=7)		
UI^b^ at baseline (n=1), n (%)		0 (0)	0 (0)	1 (100)	.99^c^	
Age (years), median (IQR)		34 (32-36)	41 (37-42)	33 (30-36)	.03^d^	
Multipara (n=14), n (%)		6 (43)	2 (14)	6 (43)	.99^c^	
BMI before pregnancy (kg/m^2^), median (IQR)		21 (19-25)	20 (19-21)	18 (17-20)	.07^d^	
Weight gain during pregnancy (kg), median (IQR)		12 (10-13)	10 (6-16)	10 (8-10)	.35^d^	
Child’s birth weight (g), median (IQR)		3213 (3060-3530)	2722 (2704-2885)	2752 (2675-2932)	.01^d^	
**Weekly mean number of PFMCs^e^ performed per day (times), median (IQR)**						
	Week 1	18 (17-18)	11 (5-12)	3 (2-7)	.004^d^	
	Week 2	17 (15-18)	13 (8-15)	2 (2-4)	.003^d^	
	Week 3	18 (17-18)	11 (9-15)	2 (4-1)	.001^d^	
	Week 4	18 (17-18)	9 (8-18)	2 (1-3)	.002^d^	
	Week 5	18 (18-18)	15 (7-15)	2 (1-3)	.001^d^	
	Week 6	18 (14-18)	8 (6-15)	2 (1-3)	.001^d^	
	Week 7	18 (17-18)	9 (6-14)	1 (1-3)	<.001^d^	
	Week 8	18 (18-18)	7 (5-8)	2 (1-3)	.001^d^	
	Total PGHD^f^ (times)	53 (43-56)	54 (46-54)	52 (40-54)	.64^d^	
“**Was it difficult to record the PFMCs every day?” (n=12), n (%)**						.17^c^
	“Strongly agree” and “Agree a little” (n=1)	0 (0)	1 (100)	0 (0)		
	“Neither agree nor disagree,” “Disagree a little,” and “Strongly disagree” (n=11)	4 (36)	1 (9)	6 (54)		

^a^PFMT: pelvic floor muscle training.

^b^UI: urinary incontinence.

^c^Fisher exact test.

^d^Mann-Whitney *U* test.

^e^PFMC: pelvic floor muscle contraction.

^f^PGHD: patient-generated health data.

### Usability of PGHD Recording

After the intervention period, some participants (27/47, 57%) answered questions about the usability of PGHD recording (Table 4). The number of responses was approximately equal for both high (15/27, 56%) and low (12/27, 44%) categories of engagement with PGHD recording. Furthermore, a significant difference between the level of engagement with PGHD recording and usability was found (*P*=.01, Fisher exact test), indicating that those who found PFMC recording difficult had low engagement with PGHD recording. The total percentage of participants who answered “Strongly agree” and “Agree a little” to the question “Was it difficult to record the PFMCs every day?” was 37% (10/27).

Uncategorized comments by the participants were as follows: “By reporting the number of times, I felt as if I was being watched for not being lazy, and I think I was able to continue,” “I did not report PFMCs, but I was able to perform them every day,” “When I receive emails three times a day, the importance of emails gradually decreased for me, and I wish I could set the number of times emails were sent individually,” and “At first, I was motivated, but I tended to skip halfway through.”

**Table 4:** Usability of patient-generated health data recording.

Response		Participants (n=27), n (%)
“**Was it difficult to record the PFMCs^a^ every day?”**		
	Strongly agree	3 (11)
	Agree a little	7 (26)
	Neither agree nor disagree	4 (15)
	Disagree a little	12 (44)
	Disagree	1 (4)
**Categorized comments**		
	“I was able to continue training by reminder email.”	14 (52)
	“It was difficult to secure time for training while raising children.”	6 (22)
	“Nothing in particular.”	3 (11)
	Other	4 (15)

^a^PFMC: pelvic floor muscle contraction.

## Discussion

### Principal Findings

In this study, we examined patients’ engagement with PGHD recording integrated into a multicomponent intervention and evaluated the impact of PGHD recording on their health behavior changes. The following findings were obtained. First, engagement with PGHD recording might be low. This could be because the number of PGHD recorders declined over time, as indicated on a sigmoid curve. Moreover, a small number of participants recorded PGHD continuously (17/47, 36%). Second, PGHD recording may not promote health behavior changes. This was suggested by the overall poor PFMT adherence observed (10/17, 59%).

### Comparison With Prior Work

Eysenbach [17] has hypothesized that fluctuations in the number of users and dropouts in digital health can be classified into Phases I-III in “sigmoid attrition curves.” Phase I is the stage where users initially stay because of curiosity, Phase II is the stage where the number of users decreases rapidly (ie, the stage where the users’ expectations are not met), and Phase III is the stage where “hardcore” users stabilize. We derived a “sigmoid attrition curve” from the obtained data, which aligned with Eysenbach’s attrition hypothesis. However, not all attrition curves for fluctuations in the number of users and dropouts in digital health are sigmoidal. The app-based intervention for diabetes prevention used by Fukuoka et al [34] for obese adults at risk for type 2 diabetes incorporated a core curriculum consisting of PGHD recording (daily steps), reminders, and face-to-face sessions, with the proportion of PGHD recorders declining in a linear function from approximately 80% at the start of the intervention to approximately 40% over the 20-week intervention period. Similarly, Carter et al [35] have reported interventions for weight loss in overweight volunteers using a smartphone app that included PGHD recording of food diaries and physical activity, sending SMS text messages to reinforce health behaviors, and feedback on the recorded physical activities in combination with face-to-face group sessions with the number of PGHD recorders declining progressively in a linear function from 43 recorders at the start of the intervention to 7 recorders (16%) over the 6-month intervention period. In either study, the attrition curve was not mathematically derived, and a sigmoid curve was not obtained. The shape and slope of these attrition curves are reportedly dependent on the age and sex of the PGHD recorders [36], the type of PGHD [37,38], the lack of relative advantages over digital health for users, usability (complexity), trial settings (such as trial management or reminders by researchers), and user attributes [17]. Possible reasons for the small number of PGHD recorders in this study include the following usability issues: 37% (10/27) of users felt that PFMC recording was burdensome, and those who found PFMC input difficult had low engagement with PGHD recording. In addition, 22% (6/27) of the users mentioned that “it was difficult to secure time for training while raising children” in the usability comments, which may be partly due to the participants of this study being postpartum women who were busy with childcare, had no time to perform PFMT, and could not record the number of PFMCs performed. In this study, PGHD recorders abruptly decreased from week 2 to week 3, which may be an appropriate time period to reinforce individualized interventions with personal support for users. In addition, since the number of participants who recorded PGHD stabilized after week 4, it is desirable to perform the intervention evaluation after this period. Thus, analyzing and understanding changes in the number of users and dropouts is important to enhance the efficacy of the intervention.

PFMT adherence in the 17 participants who recorded PGHD continuously could be clearly categorized into 3 latent classes. The moderate- and low-level classes (combined: 10/17, 59%) were considered to have poor PFMT adherence, and the weekly mean number of PFMCs performed per day was low even in PGHD recorders. In the present analysis, data that were not recorded as PGHD were treated as missing data. The comment “I did not report PFMCs, but I was able to perform them every day” by some participants suggests that some of those who did not record PGHD actually performed PFMT without recording it; thus, we considered an input of 0 for PFMCs performed as invalid. Accordingly, PFMT adherence may have been higher than shown in the data. However, as PFMT is a muscle-strengthening exercise, a minimum number of contractions and consistent practice (for at least 8 weeks) are both required. Therefore, treatment and prevention of UI cannot be expected unless the PFMT adherence pattern is similar to that of the high-level class drawn by LCGM.

Even users who showed high engagement with PGHD recording did not necessarily adhere to the PFMT regimen as instructed. These data suggest that PGHD recording may not promote health behavior changes. One of the PGHD usability comments was “By reporting the number of times, I felt as if I was being watched for not being lazy, and I think I was able to continue.” A previous study [23] reported that PGHD recording leads to positive attitudes among users, such as increased awareness of health behaviors. However, in our study, the finding that less than half of the users adhered to the PFMT regimen as instructed suggests that although PGHD recording may have a promotive effect on the users’ awareness of changing their health behavior, it might not be sufficient to promote health behavior changes. Steinberg et al [39] implemented an intervention combining recording of the previous day’s steps as PGHD, feedback on the recorded number of steps, and group sessions in women with a BMI ≥25 kg/m^2^. Of the 26 participants in the intervention group, 8 stopped recording PGHD during the intervention period, and the proportion of participants who recorded PGHD, which was approximately 80% at the start of the intervention, gradually declined to approximately 25% during the 24-week intervention period. They also reported that there was no correlation between the rate of PGHD recording during the intervention period and the number of steps as a measure of change in health behavior. Although it has been reported that PGHD recording is independent of health behavior changes, there is a possibility that intervention studies have been conducted and analyzed on the assumption that PGHD recording promotes health behaviors. Few studies have shown the impact of PGHD recording on health behavior outcomes, and the evidence is still lacking. Recently, it has been reported that effective use patterns of multicomponent interventions might differ across users, and that users do not always have to use all of the intervention elements [40]. In addition, the possibility that PGHD recording itself may lead to lower engagement for health behavior changes cannot be ruled out [9]. Based on previous studies and our results, clinicians and researchers must understand that all users who record PGHD in multicomponent interventions do not necessarily adhere to health behavior changes.

### Limitations

There are 3 limitations of this study. First, the sample size was too small to clearly demonstrate associations between PGHD recording and health behavior changes. One reason for the small sample size is that participants did not receive explicit instructions that they had to record PGHD, which was a component of the system, at the time of study participation. The participants recorded, or did not record, PGHD at their own discretion. Therefore, those who did not consider the PGHD recording of their PFMT necessary might not have continued PGHD recording. When participants use a multicomponent system, such as the one in our study, it is difficult to ensure that all components are used. Despite this limitation, this is one of the few studies that used LCGM to evaluate PFMT adherence, a measure of health behavior change, and investigated the impact of PGHD recording on health behavior change. Therefore, we believe that this case study will lead to a larger-scale survey. Second, the intervention in this study was a multicomponent intervention combining PGHD recording and PFMT reminder emails; as such, the effect of PGHD recording alone could not be evaluated. However, it is commonly accepted in PGHD research that evaluation of PGHD recording alone is difficult because it is an integral part of multicomponent interventions. In the future, a research design that can evaluate the impact of PGHD recording alone on health behavior changes needs to be established. Third, the number of PFMCs performed as a measure of PFMT adherence is a self-reported outcome and could not be confirmed. Therefore, systemic errors could occur as a result of participants reporting an inaccurate number of PFMCs performed. Given this limitation, the results must be carefully interpreted.

### Conclusions

The number of users who recorded PGHD in a multicomponent intervention declined over time in a sigmoid curve. A small number of users recorded PGHD continuously, and users felt that PGHD recording was burdensome. Therefore, PGHD engagement was found to be low. In addition, more than half of the PGHD recorders had poor PFMT adherence. These results suggest that PGHD recording may not always promote health behavior changes. Clinicians and researchers must understand that users who record PGHD in multicomponent interventions do not necessarily adhere to health behavior changes.

## Abbreviations

DBCI: digital behavior change intervention
LCGM: latent class growth modeling
PFMC: pelvic floor muscle contraction
PFMT: pelvic floor muscle training
PGHD: patient-generated health data
UI: urinary incontinence
